# A Narrative Review on Pseudocereals and Cardiometabolic Health: Biological Mechanisms and Evidence from Human Studies

**DOI:** 10.3390/nu18071093

**Published:** 2026-03-29

**Authors:** Yesim Oztekin, Zehra Buyuktuncer

**Affiliations:** Department of Nutrition and Dietetics, Faculty of Health Sciences, Hacettepe University, Ankara 06100, Türkiye; yesimoztekin@hacettepe.edu.tr

**Keywords:** pseudocereals, quinoa, buckwheat, amaranth, cardiometabolic health, human studies

## Abstract

**Background/Objectives**: Demand for functional foods is growing due to the desire to prevent cardiometabolic disorders. Pseudocereals, particularly quinoa, buckwheat, and amaranth, stand out for their functional properties related to cardiometabolic health. The dietary fiber, plant proteins, vitamins, minerals, and phytochemicals in pseudocereals primarily help to regulate glycemic response and lipid profile, as well as blood pressure. The aim of this review is to briefly explain the role of pseudocereals in biological mechanisms underlying cardiometabolic effects and evaluate the findings of human studies. **Methods:** The biological mechanisms that emphasize potential cardiometabolic effects of pseudocereals were summarized based on preclinical studies. Human studies were searched on Web of Science, PubMed, and ScienceDirect between June and December 2025. Findings of human studies on potential cardiometabolic health benefits of pseudocereals, including their anti-hyperlipidemic, anti-hyperglycemic, anti-obesity, and anti-hypertensive effects, are discussed. **Results:** The revealed mechanisms in preclinical studies and current outcomes of thirty-three human studies included in this review indicated that pseudocereals, especially quinoa and buckwheat, might be a part of healthy nutrition to assist the prevention and management of cardiometabolic disorders. In human studies, the most notable improvements were reported in plasma triglyceride and total cholesterol levels. Nevertheless, the number of human studies is limited, and existing studies have methodological variations to state cumulative and evidence-based consumption recommendations. **Conclusions:** Despite the potential protective effects of pseudocereals on cardiometabolic health, well-designed, controlled human studies are needed to elucidate the outcomes and provide clear evidence of the role of pseudocereals in relation to cardiometabolic effects.

## 1. Introduction

Wheat, rice, and maize are the most widely cultivated and consumed cereals globally. They have been constituted as the largest part of energy and carbohydrate sources in the human diet [1]. High energy and carbohydrate intake, especially sugars, have been associated with the risk of cardiometabolic disorders, including obesity, diabetes, and cardiovascular disease. In 2021, the global death from non-communicable diseases (NCDs) exceeded 17 million people under the age of 70. It was reported that the prevalence of cardiometabolic disorders is increasing globally, and obesity is the major risk factor for cardiometabolic diseases [2]. According to World Health Organization (WHO) data from 2022, 2.5 billion adults aged 18 years or older were classified as overweight. Within this group, 890 million people were living with obesity [3]. It was reported in the World Obesity Atlas 2025 that 1.45 billion adults will have a high BMI by 2030 if current trends continue. Additionally, it is predicted that obesity will lead to an economic burden of approximately $3 trillion per year by 2030 [4]. The increasing prevalence of obesity has resulted in a growing interest in dietary modifications, including consumer demand for functional foods that can support cardiometabolic health. One of the food subgroups that has functional characteristics is “Pseudocereals”. Pseudocereals, also known as “pseudo-grains,” “sub-exploited cereals,” “underutilized crops,” “Andean cereals,” or “ancient cereals,” were historically more commonly consumed by populations, particularly by the Inca, Maya, and Aztec civilizations [5]. Quinoa, buckwheat, and amaranth are the most well-known pseudocereals. “*Chenopodium quinoa*”, “*Fagopyrum tataricum*” or “*Fagopyrum esculentum*” and “*Amaranthus hybridus*” or “*Amaranthus caudatus*” are common edible botanical types for quinoa, buckwheat, and amaranth, respectively [6,7]. While pseudocereals are dicotyledons, traditional cereals such as wheat, rice, and maize are monocotyledons. Quinoa and amaranth are botanical members of the *Amaranthaceae* family, and buckwheat is a member of the *Polygonaceae* family [8]. They have higher tolerance to salinity and drought compared to traditional cereals [9,10]. Buckwheat and amaranth contribute to sustainable food systems, enhancing resilience and biodiversity. Pseudocereals also differ from traditional cereals in terms of their nutritional matrix. Macronutrients, particularly carbohydrates, are found within the endosperm structure of cereals, while they are found in perisperm in pseudocereals [11].

Considering their nutritional characteristics, pseudocereals contain higher levels of plant proteins and essential amino acids, including leucine, phenylalanine, and lysine. Especially, quinoa includes all essential amino acids, and this makes it a complete plant protein source. Their high lysine content differs from that of traditional cereals, which typically contain very limited amounts of this essential amino acid [12]. Moreover, their unsaturated fatty acids, dietary fibers, vitamins, minerals, polyphenols, and phytosterols contents are higher than those of traditional cereals [8,12,13]. Therefore, pseudocereals have been studied for their potential anti-hyperlipidemic, anti-hyperglycemic, anti-hypertensive, and anti-obesity effects on cardiometabolic health due to their nutrient and bioactive components [14].

Despite the growing body of evidence about the effects of pseudocereals on cardiometabolic mechanisms, findings depend on preclinical studies, and the results of in vitro or animal research are not adequate to develop dietary strategies for humans. Existing reviews generally underlined the nutritional and functional potential of pseudocereals by focusing on the outcomes of in vitro or animal studies [7,15]. However, the results of in vitro and animal research cannot be precisely reflected in humans. It is known that metabolic pathways in the human body are more complex, and many environmental factors can change the functions of human biological systems. These factors restrict the strength of results as well as decrease their clinical applicability to human health. Hence, the evaluation of the findings of human trials has gained importance so as to reveal the health benefits of pseudocereals on the human body. With this context, the potential impacts of pseudocereals on cardiometabolic health have been evaluated in this review. Then, human studies were critically assessed, and the impacts of pseudocereals on cardiometabolic health were synthesized to determine who can experience the therapeutic effects of pseudocereals and which conditions can produce these effects. Consequently, the present review indicates this point and aims to summarize the potential roles of pseudocereals in cardiometabolic mechanisms and to subsequently assess the findings of human studies to provide an implication on the benefits of pseudocereals for human cardiometabolic health.

### Potential Mechanisms of Pseudocereals on Cardiometabolic Health

The potential anti-hyperlipidemic effects of pseudocereals depend on changes in gene expression in lipid storage, such as 3-hydroxy-3-methyl-glutaryl-coenzyme A-reductase (HmG-CoA reductase), farnesyl-diphosphate farnesyltransferase-1 (FDFT-1), and squalene monooxygenase (SQLE), a decrease in endogenous cholesterol synthesis, an increase in fecal excretion of bile salt and cholesterol [16], inhibition of lipid peroxidation with their bioactive components such as 20-Hydroxyecdisteroids, fagopyritols, and betacyanin [17,18,19,20], and regulation of intestinal microbiome [21].

The second cardiometabolic health-improving mechanism is related to the anti-hyperglycemic effects of pseudocereals. The food composition of pseudocereals, including a high content of plant protein, bioactive peptides, dietary fiber [22,23], and phytochemicals [8,24], and hence a low glycemic index [25], regulates glycemia. They perform these effects by protecting cells against oxidative stress and preventing insulin receptor damage [24,26], producing short-chain fatty acids (SCFA) [27], inhibiting α-amylase and dipeptidyl peptidase-IV (DPP-IV) enzymes [28], delaying glucose absorption [29], and correspondingly, regulating insulin and glucose levels.

Pseudocereals also possess anti-obesity properties through mechanisms associated with suppression of appetite and longer satiety duration [30,31], a reduction in obesity-related inflammation and oxidative status [32,33,34,35], an increase in energy expenditure and inhibition of adiposity [36], and regulation of intestinal microbiome [37]. Soluble fiber, resistant starch, and undigestible polysaccharides such as xyloglucans and pectic polysaccharides provide a low glycemic index, prolong satiety, reduce food intake, and contribute to daily energy balance [31,38]. It has been shown that the protein extracts or hydrolysates of pseudocereals can activate the peroxisome proliferator-activated receptor-γ (PPAR-γ) pathway and inhibit adipocyte proliferation and lipid accumulation in the body [36]. Therefore, protein fractions in pseudocereals can help to reduce the risk of obesity.

**Figure 1:**
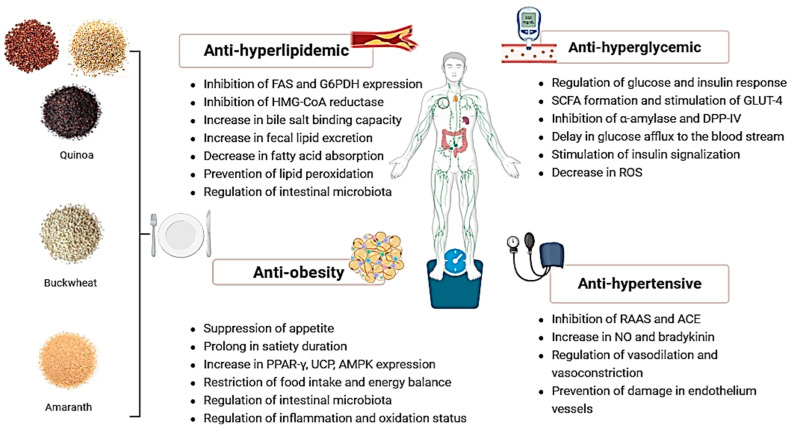
Functional roles of pseudocereals on cardiometabolic health. Abbreviations: ACE, angiotensin-converting enzyme; AMPK, adenosine monophosphate-activated protein kinase; BMI, body mass index; BP, blood pressure; DPP-IV, dipeptidyl peptidase-IV; FAS, fatty acid synthase; G6PDH, glucose-6-phosphate dehydrogenase; GLUT-4, glucose transporter type 4; HbA1C, Hemoglobin A1c; HDL, high density lipoprotein; HMG-CoA, 3-hydroxy-3-methyl-glutaryl-coenzyme A-reductase; LDL, low density lipoprotein; NO, nitric oxide; RAAS, renin–angiotensin–aldosterone system; RCTs, randomized controlled trials; PPAR-ɣ, Peroxisome Proliferator-Activated Receptor Gamma; ROS, Reactive Oxygen Species; SCFA, short-chain fatty acid; UCP, Uncoupling Protein.

The other crucial component of cardiometabolic health is blood pressure. Inhibition of renin–angiotensin–aldosterone system (RAAS) [39] and angiotensin-converting enzyme (ACE) activity [40,41,42], regulation of vasodilation and vasoconstriction [43,44], and prevention of damage in endothelium tissue of vessels [45] are possible mechanisms for anti-hypertensive effects of pseudocereals. It was emphasized in a current literature review that amaranth protein hydrolysates and isolates have regulatory effects on blood pressure by inhibiting RAAS activation [39]. Bioactive peptides in amaranth and quinoa inhibit ACE through interaction between specific amino acid chains and ACE [41,42]. The inhibition of ACE provided an increase in bradykinin and endothelial nitric oxide (NO) levels and supported the regulation of blood pressure [43,44]. Additionally, quercetin and rutin in buckwheat performed a regulatory role in blood pressure. Rutin in buckwheat decreases oxidative damage of the endothelium in vessels and strengthens the capillary wall against high blood pressure [45]. The results of the studies indicate that bioactive compounds found in pseudocereals have anti-hypertensive effects. Figure 1 shows the proposed biological mechanisms underlying the cardiometabolic effects of pseudocereals. In the following sections of this review, current findings from human studies will be discussed. In general, research focuses on the health-improving effects of pseudocereals rather than solely weight reduction. Most preclinical studies implicated that the anti-hyperglycemic and anti-hyperlipidemic potential of pseudocereals may bring together anti-obesity effects. The anti-obesity properties of pseudocereals may occur as a secondary effect following their anti-hyperlipidemic and anti-hyperglycemic actions.

Pseudocereals may also show these cardiometabolic health-improving effects through modulation of the intestinal microbiome and brain–gut–microbiome axis [21,46,47]. Variations in gut microbiome composition may be related to the bioactive components and high fiber content of pseudocereals [46,47]. Quinoa significantly increased the abundance of *Enterococcus*, *Turicibacter*, and *Akkermansia* in obese mice. The increased abundance of these genera made the gut microbiome more similar to that of lean mice [48]. The pathway between pseudocereal-based microbiome alterations and cardiometabolic impacts was associated with the gut–brain axis. Short-chain fatty acids (SCFAs), particularly butyrate and propionate, produced by intestinal microbiota, may stimulate the secretion of GLP-1 and PYY. GLP-1 and PYY may send satiety signals to the hypothalamus and, correspondingly, may decrease food intake and support energy balance [49,50]. Despite growing interest in the effects of pseudocereals on gut microbiome, findings examining their effects on the intestinal microbiome and brain–gut–microbiome axis are still limited. The validation of these findings requires well-designed human trials. The other characteristics of pseudocereals, including quinoa, buckwheat, and amaranth, are rich in minerals, especially compared to traditional cereals. They include important amounts of magnesium, potassium, iron, zinc, calcium, and phosphorus, and increase dietary intake of these minerals. The mineral contents of quinoa, buckwheat, and amaranth provide high potassium and magnesium intake, which support cardiometabolic health by regulating blood pressure and alleviating insulin resistance [51,52]. On the other hand, they include some anti-nutrients such as saponin, phytate, and oxalates. These anti-nutrients bind some cations, such as Fe, Zn, Ca, Mg, and lower their bioaccessibility [53,54]. 

## 2. Materials and Methods

### Review Strategy for Human Studies

**Figure 2:**
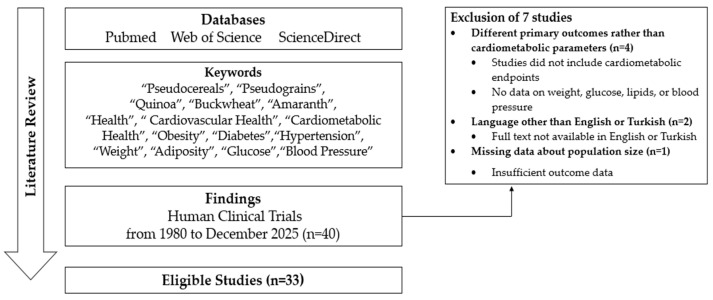
Flow chart for the literature review.

While this review is a narrative review, a structured search plan was used in methodology. Web of Science, PubMed, and ScienceDirect databases were reviewed between June 2025 and December 2025 to analyze and synthesize related human clinical studies. “Pseudocereals,” “pseudograins”, “quinoa”, “buckwheat”, “amaranth”, “health”, “cardiovascular health”, “cardiometabolic health”, “obesity”, “diabetes”, “hypertension”, “weight”, “adiposity”, “glucose” and “blood pressure” keywords and their combinations were used. Search strings used for each database were given as: (“pseudocereal*” OR quinoa OR amaranth OR buckwheat) AND (“cardiometabolic” OR “cardiovascular risk” OR lipid* OR “glycemic control” OR “obesity” OR “weight” or “blood pressure”). Then, the results were filtered by “Human” research. A brief flow chart of the eligible studies is given in Figure 2. Human observational and interventional studies that investigated the cardiometabolic effects of pseudocereals on lipid profile, blood glucose parameters, blood pressure, or anthropometric indices, conducted between the years 1980 and 2025, were included. The year 1980 was selected as the starting point to ensure comprehensive coverage of the literature because the related studies were being published more around this time. Additionally, findings from meta-analyses of human trials were given for cumulative evidence of high quality. Manuscripts published in languages other than Turkish and English, or not fully published manuscripts, were excluded. On the other hand, in vitro and animal studies, unpublished reports, book chapters, editorials, abstracts, studies investigated different primary outcomes, and studies reporting missing data were excluded.

Keywords, strings, and all inclusion and exclusion criteria were determined by both authors. Then, study selection was performed by the primary author, considering inclusion and exclusion criteria. The discussion and consensus method was used with the participation of a third independent reviewer in case of disagreements between authors. To prevent selection bias, inclusion and exclusion criteria were strictly defined before the literature search. A total of 40 human clinical trials were identified through database searches. After screening and eligibility assessment, 7 studies were excluded due to different primary outcomes (n = 4), language other than English or Turkish (n = 2), or missing population size information (n = 1). Finally, 33 studies were included in the narrative synthesis.

## 3. Results

After the literature review, forty human clinical studies were recorded. However, seven studies were excluded due to different primary outcomes, languages other than English or Turkish, and missing data. The thirty-three studies included in this literature review are summarized in Table 1. The main findings were evaluated by considering variations in studies, such as the type of pseudocereals, study population, intervention (if applicable), and comparison factors, to reach a current implication from human studies. 

### 3.1. Anti-Hyperlipidemic Effects Reported in Human Studies

#### 3.1.1. Quinoa

The methodological evaluation of studies in different populations, as well as their findings help to explain which populations experience the anti-hyperlipidemic effects of pseudocereals. Consumption of quinoa biscuit reduced serum total cholesterol (TC) and serum low-density lipoprotein–cholesterol (LDL-C) compared to wheat biscuit in individuals aged between 50 and 75 years [55]. In another study conducted with a young adult population, a cereal bar containing 19.5 g of quinoa provided a 10% decrease in serum TC, 12% in serum triglycerides (TG), and 21% in serum LDL-C levels [56]. The findings of these two studies support that quinoa consumption has the potential to improve the lipid profile in adults. Following the consumption of 25 g/day of quinoa or corn flakes in postmenopausal women, serum LDL-C and TC decreased by 6% and 5%, respectively, in the quinoa group. In the quinoa group, an increase in the Glutathione-S-Transferase (GSH) level helped metabolize lipid peroxidation molecules and prevent endothelial dysfunction [57]. The decrease in cholesterol levels is associated with the higher content of fiber and antioxidant compounds of quinoa compared to corn. It was found that consumption of 25 g/d of quinoa for 12 weeks significantly decreased only the serum TG levels, while 50 g/d of quinoa decreased the prevalence of metabolic syndrome in overweight and obese participants [58]. The higher amount of quinoa consumption provided higher dietary fiber and antioxidant compound intake and resulted in relatively more distinct changes to prevent metabolic syndrome parameters. It can be emphasized that observations through this study indicate that the amount of daily pseudocereal consumption is another critical point for assessing their anti-hyperlipidemic effects. A meta-analysis of five studies, with quinoa consumption ranging from 20 to 65 g/day, concluded that quinoa can decrease serum TG, TC, and LDL-C levels [88]. Another meta-analysis of human studies found that higher than 50 g/d of quinoa and an intervention period longer than 6 weeks significantly provided a reduction in serum TG levels, whereas there were no significant changes in serum TC, LDL-C, and HDL-C [89]. These meta-analyses have reached a common conclusion regarding the positive effects of pseudocereal consumption on TG levels. However, the accurate dose and duration for therapeutic effects were not specified precisely.

#### 3.1.2. Buckwheat

Pseudocereals are underutilized crops; therefore, their habitual intake is restricted in a limited number of regions, such as South Africa, Latin America, Central Asia, China, and Mongolia [13]. Two cross-sectional population-based studies reported that serum TC and LDL-C levels were lower in people consuming buckwheat [59,60]. Habitual buckwheat consumption can contribute to a decrease in the prevalence of hypertriglyceridemia and hyperlipidemia [59]. Furthermore, the average consumption of 100 g/d of buckwheat for one year provided an increase in the ratio of HDL-C to TC, which is currently a more reliable parameter for cardiometabolic health [60]. These findings indicate that long-term and regular consumption of buckwheat is associated with a decrease in the prevalence of hyperlipidemia. It can be highlighted through all these studies that regular pseudocereal consumption is another critical point when observing its anti-hyperlipidemic effects. Two similar studies conducted with buckwheat bread showed a decrease in serum TC and LDL-C levels and an increase in the HDL-C/TC ratio [61,62]. These improvements in lipid profile were attributed to the varied fiber and flavonoid content of buckwheat. Cookies made with both *Fagopyrum tataricum* and *Fagopyrum esculentum* decreased serum TC levels in healthy people; however, serum myeloperoxidase levels declined only in the people consuming *Fagopyrum tataricum* group [63]. The reason for this difference between buckwheat species can be related to the higher bioactive components of *Fagopyrum tataricum* [90]. Correspondingly, this finding is consistent with the results of the human study conducted by Wieslander et al., which also showed the higher anti-hyperlipidemic potential of *Fagopyrum tataricum* [63]. In addition to promising outcomes of buckwheat in healthy populations, serum cholesterol and glucose-lowering effects of buckwheat were reported in different diseases such as metabolic syndrome, type 2 diabetes mellitus (T2DM), and renal disease [64,90]. In individuals with mild or moderate hypercholesterolemia, buckwheat-fortified protein porridge lowered serum TC, LDL-C, TG, and uric acid levels compared to corn-fortified porridge. Moreover, buckwheat-fortified protein porridge increased serum adiponectin, HDL-C, and fat-free mass [90]. It can be said that the replacement of traditional cereals with pseudocereals can be an alternative way to create a healthy diet in terms of lipid profile. Substitution of one portion of refined rice or wheat with buckwheat reduced serum TC, LDL-C, and fasting insulin levels [65]. The outcomes of these studies confirm the nutritional advantages of buckwheat. Similarly, a meta-analysis reported that a higher consumption of buckwheat is associated with slight but favorable changes in blood lipid and glucose levels [91].

#### 3.1.3. Amaranth

Amaranth is less commonly consumed than pseudocereals compared to buckwheat or quinoa due to its unfamiliar taste and texture, has limited processing capacity, and limited commercial support. This situation correspondingly restricted the number of human studies on the potential effects of amaranth. There are only five studies evaluating the anti-hyperlipidemic effects of amaranth in humans. Additionally, while quinoa and buckwheat are primarily studied in grain form, amaranth has mostly been investigated as amaranth oil due to its concentrated tocopherols, phytosterols, and squalene content [92]. Previous controlled interventions found that there are no effects or mild favorable impacts of amaranth oil on TC and LDL-C. Two studies showed that amaranth oil can help to regulate cardiac rhythms in people with arrhythmia or hypertension [66,93]. In contrast, Jamka et al. reported that there were no significant differences between 20 mL/d of amaranth oil and rapeseed oil on atherosclerosis markers of overweight or obese people [67]. On the other hand, substituting amaranth oil for rapeseed oil interestingly elevated cardiovascular risk in a study on overweight and obese individuals [68]. These inconsistent results reflect study heterogeneity. Variations in study population, habitual diet, daily dose, and duration of amaranth oil and small sample sizes are key reasons for variation. It can be implemented from these conflicted findings that the lipid-lowering effect of amaranth oil is, first, dose-dependent; second, influenced by the characteristics of the population; and finally, the choice of control group may be a decisive factor in results.

Briefly, the results of human studies show that pseudocereals may have mild benefits on lipid profile when they are used instead of refined carbohydrates or unhealthy fats in the diet. However, these benefits are not clinically significant. In people who require larger guideline-recommended LDL reductions, pseudocereal supplementation is not adequate to observe clinical improvements in any lipid profile. Heterogeneity across studies differing in products, doses, durations, background diets, and small sample sizes likely explains conflicted findings and emphasizes the need for larger, standardized RCTs. Consequently, the effects of pseudocereals on lipid profiles in clinical practice have not been explained clearly yet.

### 3.2. Anti-Hyperglycemic Effects Reported in Human Studies

The effects of pseudocereals on blood glucose levels show variety in the human body, based on their botanical types or production, processing, preparation, and cooking techniques, as well as unique differences between individuals. Furthermore, food processing techniques—including milling, extrusion, fermentation, germination, and different cooking methods—can substantially modify starch gelatinization, resistant starch formation, and the structural integrity of the food matrix, thereby affecting carbohydrate bioavailability and glycemic impact [94].

Previous studies have shown that even when the same pseudocereal species is used, variations in processing conditions, food matrices, portion sizes, and preparation methods may lead to different glycemic outcomes [94,95]. In addition, heterogeneity in study designs and participant characteristics further contributes to the differences in reported results. Therefore, the current body of literature does not yet allow a standardized classification of these factors. For this reason, the revised section discusses these influences narratively rather than presenting them in a simplified table, in order to avoid oversimplifying the complex interactions that affect glycemic responses. Common botanical types of pseudocereals or food production and processing techniques affect cardiometabolic health (Table S1). For example, phenolic compounds show a variety in white, red, and black quinoa or *Fagopyrum tataricum*, which contains more phenolic compounds than *Fagopyrum esculentum* [63]. In a similar way, raw buckwheat grains have more bioactive compounds than grounded buckwheat grains because it leads to decreased bioactive compounds. Additionally, some studies show that amaranth can show its cardiometabolic health effects in oil form. All these factors may change the physicochemical characteristics of pseudocereals, change digestibility, antioxidant capacity, and, correspondingly, blood glucose levels.

#### 3.2.1. Quinoa

A quinoa-based diet reduced postprandial glucose levels in the prediabetic geriatric population, and substitution of carbohydrate sources with quinoa decreased carbohydrate intake and increased lipid and protein intake [69]. These results can be primarily attributed to the high plant protein content in quinoa and its synergistic effects with dietary fiber and phenolic compounds that delay carbohydrate digestion. Moreover, this study reveals that the health-improving effects of quinoa can be observed in cardiometabolic disease risk groups such as older adults. However, studies that have a larger sample size are required to obtain more robust results. Different outcomes were reported considering gender, where an 8% decrease in glucose concentration was observed in males following 30 days of quinoa consumption [56]. Higher muscle mass and a lower fat percentage in men may increase glucose use in muscle tissue. Therefore, low-glycemic index foods like quinoa may regulate blood glucose levels more quickly and noticeably in men. A comparison of the glycemic responses showed that bread containing 20% quinoa flour produced a significantly lower cumulative area under the blood glucose curve than bread made from 100% refined wheat flour [70]. It is predicted that insoluble fiber content and lower available carbohydrates in quinoa bread delay glucose afflux to the bloodstream and provide balance in glucose levels. Similarly, consumption of 100 g/d of bread containing 20% quinoa flour significantly decreased blood glucose [71]. The number of studies conducted on long-term consumption is limited. Two studies with a one-year follow-up, which looked at quinoa consumption and improved glycemic control and lipid profile, resulted in a significantly lower progression risk from impaired glucose tolerance to diabetes in the quinoa group [72,73]. These studies are prominent in observing the impacts of long-term quinoa consumption on glycemic regulation and the prevalence of T2DM. It can be inferred that the inclusion of quinoa in the diet for a long period can demonstrate therapeutic effects against diabetes mellitus. Pseudocereals have been added to new food formulations to enhance the functional properties of food products. For example, fermented quinoa-based blackcurrant products provided more balanced glucose levels and prevented fluctuations in postprandial glucose and insulin levels compared to blackcurrant products without fermented quinoa [74]. In this study, the low-glycemic index value or fiber content of quinoa may balance the glycemic and insulinemic impacts of blackcurrant products. Moreover, pseudocereals do not contain gluten, and they have nutrient-rich profiles compared to other gluten-free traditional cereals such as corn and rice. For cumulative results on quinoa, a meta-analysis indicated that dietary supplementation of quinoa significantly reduced insulin levels and improved metabolic syndrome components [88].

#### 3.2.2. Buckwheat

Several studies showed anti-hyperglycemic effects of buckwheat. Two clinical trials reported more balanced postprandial glucose and insulin levels following the consumption of buckwheat [65,76]. A comparison of buckwheat pasta and corn pasta indicated that buckwheat pasta improved postprandial glucose levels in type 1 diabetes and celiac disease. It is emphasized that buckwheat pasta may decrease the risk of early postprandial hypoglycemia and prevent hyperglycemia in the prolonged postprandial phase [78]. However, a study conducted in a healthy population reported conflicting results. A gluten-free pasta made from quinoa and corn flour showed a slightly high postprandial glycemic response compared to pasta made from only wheat flour or corn and rice flour [79]. The different findings of the last two studies highlight the importance of study population characteristics. The therapeutic effects on blood glucose levels can be observed more rapidly in the diabetic population. The key points are related to characteristics of the study population and the type and amount of pseudocereals consumed. Further research is needed to determine the most efficient consumption pattern to observe the anti-diabetic effects. It was known that high glucose and insulin levels result in micro- and macrovascular complications in diabetes mellitus. A diet including pseudocereals can be a useful strategy to prevent renal complications in people with T2DM. With this approach, 100 g/d of buckwheat provided a significant decrease in urine albumin creatinine ratio (UACR) and urea nitrogen (UN) levels compared to rice or wheat in type 2 diabetes patients [80]. The replacement of traditional cereals with pseudocereals can be a part of medical nutrition therapy in preventing renal complications in people with T2DM. Nevertheless, a systematic review and meta-analysis showed no significant effects of pseudocereals on diabetes mellitus despite their positive effects on glycemic control and lipid profile [96]. The fundamental limitation of this meta-analysis is the heterogeneity of studies in terms of grain types, consumption dosages, and follow-up durations. Hence, these limitations may have hindered to observe expected results.

Short-term studies are notably conducted to determine postprandial glucose and insulin changes. A study conducted on bread made with buckwheat and quinoa showed better glycemia compared to white wheat bread. Both quinoa and buckwheat provided a gradual decrease in serum glucose levels, while a rapid decrease was observed in the white wheat bread group. At the time between 270–330 min, graphical curves for glucose exhibited more balanced and stable progression in quinoa and buckwheat groups compared to white wheat bread [75]. Although these findings support the benefits of quinoa and buckwheat consumption in the regulation of postprandial glucose response in humans, Stringer et al. stated that the consumption of buckwheat for one week did not have any impact on fasting glucose, lipids, or apolipoproteins in either healthy people or people with T2DM [77]. The reasons for these unexpected outcomes can include significant differences in age (37.3 vs. 60.8 years) and the BMI (23.5 vs. 32.4 kg/m^2^) value between research groups, as well as a shorter period of buckwheat consumption in the study.

#### 3.2.3. Amaranth

The number of studies on amaranth is less than the number of studies on quinoa or buckwheat. The snack bar that included 90% amaranth indicated the lowest glycemic index and glycemic load compared to a snack bar made with oat or 47.98% amaranth in healthy people [81]. In another study, both a calorie-restricted diet and only amaranth oil or rapeseed oil supplementation decreased anthropometric measurements. However, blood lipid, glucose, and insulin levels improved in only the amaranth oil group [82]. It is noteworthy that a reduction in anthropometric measurements is primarily related to negative energy balance in the human body. Consequently, the changes in anthropometrical measurements can be observed following a specific period of intervention.

### 3.3. Anti-Obesity Effects Reported in Human Studies

#### 3.3.1. Quinoa

Although there are conflicting findings noted in human studies, the anti-obesity impacts of pseudocereals were reported, especially for the overweight and/or obese population. Research found that quinoa intake is associated with weight loss and a decrease in BMI [55]. Phenolic compounds, fiber, and bioactive peptides in quinoa may stimulate the gastrointestinal hormones GIP or GLP-1, suppress appetite, and promote satiety. Following equal amounts of quinoa and rice consumption, satiety index scores are higher in the quinoa group. However, a similar energy intake was recorded in an ad libitum meal [83]. A limitation of ad libitum meal studies is that the availability of various foods may be a triggering factor that is associated with excess energy intake. Therefore, participants may consume food even when they do not feel physically hungry. The other limitation of this study is the small sample size. Additionally, quinoa cookies supported carbohydrate metabolism in intestinal microbiota compared to a high-fat/high-sugar cookie [87].

#### 3.3.2. Buckwheat

Buckwheat groats or bread made from buckwheat flour did not stimulate appetite-related sensations or food intake compared with a snack made from corn or rice flour [84]. The complex biopsychosocial pattern of appetite, including hedonic eating and environmental factors, may increase food intake and interfere with the accurate assessment of pseudocereals’ effects on appetite; furthermore, lipid peroxidation and oxidative stress are noted in the pathogenesis of obesity. Consumption of Tartary buckwheat provided a lower body weight and BMI as well as a decrease in Thiobarbituric acid reactive substance (TBARS) levels, which represent the body’s lipid peroxidation status [85]. Similarly, amaranth oil supplementation with a calorie-restricted diet improved antioxidant status in the body [86]. These studies indicate that pseudocereals can help to decrease oxidative status in obesity. It is known that high-glycemic index foods show shorter satiety duration, induce high calorie intake, and increase the tendency to gain weight. On the other hand, a low-glycemic index food prevents fluctuations and provides a steady state in blood glucose.

#### 3.3.3. Amaranth

The low-glycemic index of amaranth contributes to alleviating anabolic hormone secretion, such as insulin, cortisol, and growth hormone, and helps to decrease adiposity [81]. Despite these promising findings for the anti-obesity potential of pseudocereals, the multifactorial etiology of obesity complicates precise outcomes. A meta-analysis found that there is no significant effect of buckwheat on BMI, and it emphasized the importance of total energy and nutrient intake rather than the intake of specific foods or nutrients to manage body weight [70].

### 3.4. Anti-Hypertensive Effects Reported in Human Studies

#### 3.4.1. Quinoa

Although the anti-hypertensive effect can be explained through mechanisms, including the regulation of the renin–angiotensin–aldosterone system (RAAS), increased nitric oxide (NO) bioavailability, and strengthened vascular resistance [20,91], the effects of pseudocereals on blood pressure have been comparatively less investigated in human studies.

Considering the existing publications, three human studies investigated the effects of quinoa on blood pressure [55,56,69]. However, Pourshahidi et al. did not report any significant differences in blood pressure, either between the baseline and final values or between groups [55]. The reasons for these inconsistent results might involve an insufficient dose or duration, as well as product variability.

#### 3.4.2. Buckwheat

Despite a growing interest in buckwheat (*Fagopyrum esculentum* Moench; *F. tataricum* Gaertn.) as a functional food, evidence from human studies specifically addressing anti-hypertensive effects of buckwheat is very limited. 

Considering the inclusion and exclusion criteria of this review, a cross-sectional investigation conducted with 850 people in China reported that a buckwheat intake higher than 100 g/day was associated with reductions in blood pressure; however, this reduction was not statistically significant [60].

#### 3.4.3. Amaranth

Human studies on the anti-hypertensive potential of amaranth are very scarce. Martirosyan et al. found that the replacement of sunflower oil with the use of 6, 12, or 18 mL/day of amaranth oil for three weeks decreased blood pressure by 18%, 19%, and 21%, respectively [66].

The reason for the limited number of studies on the anti-hypertensive effects of pseudocereals may be associated with the pharmacological treatment of hypertension. Hypertension generally requires pharmacological treatment of hypertension [97,98]. Pharmacological treatment may overshadow the anti-hypertensive effects of pseudocereals or make it difficult to understand the primary reason for changes [8,99]. This situation is one of the reasons that restricts studies on hypertension and pseudocereals. Although pseudocereals may have the potential to regulate blood pressure, human clinical studies are scarce regarding their anti-hypertensive effects.

Taken together, findings of all research on anti-hyperlipidemic, anti-hyperglycemic, anti-obesity, and anti-hypertensive effects of pseudocereals have been considered; they have potential benefits on cardiometabolic health (Table S2). If all studies are stratified in terms of duration, short-term studies primarily indicate improvements in lipid parameters and postprandial glucose levels (Table S3). Reductions in serum TC, LDL-C, and TG levels were frequently reported, particularly with buckwheat-based interventions. Acute and intervention periods, especially conducted to observe postprandial glucose and insulin responses, similarly showed alleviation in fluctuations of glucose and insulin levels and decreased AUC glucose and insulin values. However, changes in anthropometric measurements and long-term glycemic indices, such as HbA1c, did not display improvements in most of the studies. The results of interventional studies between 4 and 12 weeks showed anti-hyperlipidemic impacts, especially a decline in serum TC and LDL-C levels. Attenuation in insulin resistance was more prominent among risk populations, such as people with diabetes or impaired glucose tolerance, and although decreases in body weight, BMI, waist circumference, and blood pressure were reported, these findings vary according to the characteristics of studies. On the other hand, current evidence is insufficient to determine a daily intake value for pseudocereals. The dose that maintains cardiometabolic health has not been determined because the functional dose depends on age, gender, general health, and other population characteristics. Therefore, the effective consumption value is unknown for different groups. Moreover, effective dose and response relationships should be evaluated using standardized consumption units. Hence, common units such as g/day facilitate to compare the findings. However, studies are still conducted in different units, and this situation makes it difficult to reach cumulative inferences (Table S4). In these conditions, pseudocereals can be discussed within the context of integration into dietary patterns. Consequently, data on cardiometabolic benefits of pseudocereals became more distinct, but still, there are controversial findings, particularly regarding clinical significance. Long-term interventions may indicate more robust results. Especially, observational studies that assess habitual consumption in some specific populations reported decreases in HbA1c and HOMA-IR. Furthermore, a reduction in BMI, waist circumference, and body composition may be observed in longer interventional studies. These findings support that sustainable and habitual pseudocereal consumption provides more prominent changes in cardiometabolic health.

## 4. Possible Adverse Effects of Pseudocereals

Pseudocereals are generally accepted as nutritious and safe. However, their safety may be related to IgE-mediated allergic reactions in predisposed individuals. Clinical signs range from mild symptoms such as urticaria to severe systemic reactions such as anaphylaxis [100,101]. Especially, buckwheat has potential allergens in people with atopic characteristics [100]. Therefore, while pseudocereals are safe for most people, they may lead to an unexpected health effect, notably in susceptible individuals.

## 5. Conclusions and Future Perspectives

The growing demand and an increasing body of evidence on the preventive effects of pseudocereals on cardiometabolic risk factors have made them more important in the field of nutrition. Preclinical outcomes reveal that pseudocereals may play a role in cardiometabolic mechanisms through their fiber content, bioactive peptides, unsaturated fatty acids, and phytochemical components. Moreover, preclinical experimental studies provide a bridge for human studies and help us understand the biochemical pathways. Following human study findings, clinically significant outcomes can be observed, and this holistic approach, from mechanism to clinical practice, strengthens future dietary recommendations for pseudocereals. Notably, quinoa and buckwheat may serve as alternatives to traditional cereals such as rice, maize, and wheat due to their higher nutritional value. The most prominent effects of pseudocereals were observed on lipid parameters such as serum TG, LDL-C, and TC levels. The median intervention duration of human studies included in this review was four weeks. The longer period of consumption is recommended to clarify the anti-hyperglycemic and anti-obesity effects of pseudocereals. However, it can be clearly understood that the changes in anthropometrical measurements depend on daily total energy intake rather than pseudocereal consumption. Therefore, pseudocereals may be more effective when they are used within the framework of healthy nutrition patterns.

**Figure 3:**
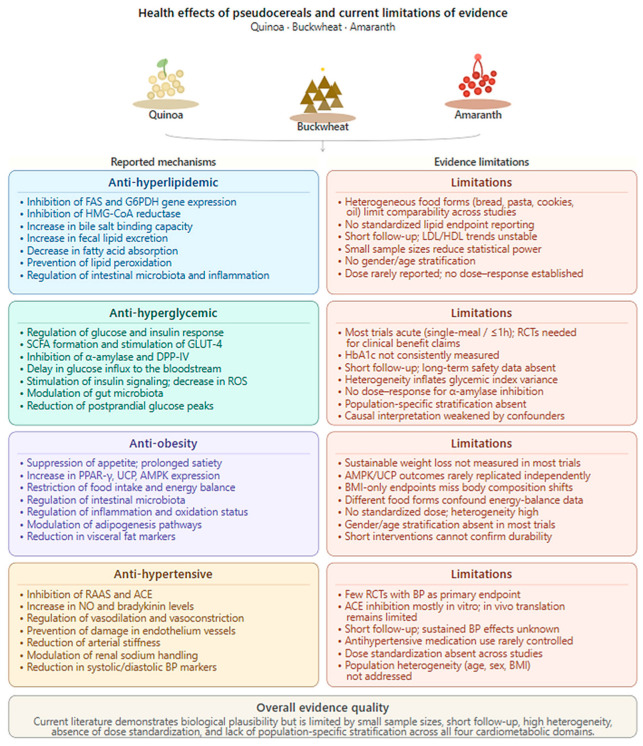
Summary of key findings and limitations in literature. Abbreviations: ACE, angiotensin converting enzyme; AMPK, adenosine monophosphate-activated protein kinase; BMI, body mass index; BP, blood pressure; DPP-IV, dipeptidyl peptidase-IV; FAS, fatty acid synthase; G6PDH, glucose-6-phosphate dehydrogenase; GLUT-4, glucose transporter type 4; HbA1C, Hemoglobin A1c; HDL, high density lipoprotein; HMG-CoA, 3-hydroxy-3-methyl-glutaryl-coenzyme A-reductase; LDL, low density lipoprotein; NO, nitric oxide; PPAR-ɣ, Peroxisome Proliferator-Activated Receptor Gamma; RAAS, renin–angiotensin–aldosterone system; RCTs, randomized controlled trials; ROS, Reactive Oxygen Species; SCFA, short-chain fatty acid; UCP, Uncoupling Protein.

In this literature review, it was realized that studies have common limitations. Figure 3 shows key findings and limitations in the literature. Considering the studies included in this review, inadequate stratification by individual characteristics, such as gender and age, restricts the ability to observe health responses specific to the population. Furthermore, adverse effects are rarely explained in clinical studies. Heterogeneity between included studies decreases the accuracy of findings. Although several studies report anti-hyperglycemic, anti-hyperlipidemic, anti-obesity, and anti-hypertensive impacts of quinoa, buckwheat, and amaranth, these studies are limited by small sample sizes, short follow-ups, and standardized food products. These factors increase heterogeneity and result in difficulties with reaching accurate inferences. A short-term follow-up restricts statistical power, and it prevents observing outcomes, safety, and sustainability in long-term effects. Additionally, some variables, such as HbA1c or sustainable weight loss, can be observed in longer interventions. The acute studies included in this review notably reflected postprandial responses (single-meal or ≤1-h designs), whereas randomized controlled trials are needed in terms of clinical benefits. An increase in heterogeneity leads to difficulties obtaining accurate inferences. Several studies lack standardized reporting of doses (grams/day). This limitation prevents determining a dose–response relationship. Taken together, the current literature shows cardiometabolic health-improving effects. However, the literature findings are limited in their high-quality clinical evidence in terms of cardiometabolic benefits of pseudocereals.

All in all, pseudocereals may help to prevent and manage cardiovascular disease, diabetes, and obesity. Focusing on a single pseudocereal, such as quinoa, buckwheat, or amaranth, may reduce heterogeneity, but this approach also means more human studies are required. The number of human studies, especially for amaranth, is inadequate to make an inference about consumption recommendations for cardiometabolic health. Therefore, the validity and reliability of current findings need to be confirmed through further human studies. While the promising effects of pseudocereals on cardiometabolic health suggest their potential inclusion in a healthy diet, well-designed and controlled human clinical trials are still necessary to establish evidence-based recommendations.

### Take-Home Messages

- Quinoa, buckwheat, and amaranth have strong mechanistic potential for cardiometabolic health.
- The integration of pseudocereals within a healthy dietary pattern may increase their functional impacts.
- Findings from human studies support translational research; however, the evidence is still heterogeneous when wanting to establish a clinical recommendation.

## Figures and Tables

**Table 1:** Characteristics and findings of human studies on cardiometabolic effects of pseudocereals.

Refs.	Population	Dose of Intervention	Duration	Main Findings
[55]	40 healthy people (F:12 M:28) Mean age: 57, 68	15 g/d of quinoa biscuit (60 g quinoa flour/100 g) 15 g/d of 100% wheat biscuit	28 days	↓ TC, LDL-C, and TC: HDL levels in the quinoa group ↓ Body weight and BMI ↓ Blood pressure No differences in dietary intake and anthropometric measurements
[56]	22 healthy students (F:13, M:9) Aged from 18 to 45 years BMI > 25 kg/m^2^ in 52% of participants	Two quinoa bars (9.75 g quinoa in each bar) in a day	30 days	↓ TC, LDL-C, and TG ↓ Glucose levels only in men. ↓ Body weight ↓ Blood pressure
[57]	35 postmenopausal women Mean age: 61 years BMI > 18.5 kg/m^2^	25 g/d of quinoa flakes (QF) (n = 18) 25 g/d of corn flakes (CF) (n = 17)	4 weeks	↓ LDL, TC, and TG in the quinoa flakes group ↑ GSH ↓ TBAR and Vitamin E in both groups ↑ Excretion of enterolignans in urine
[58]	50 overweight or obese people (F:34 M:16) Aged from 18 to 65 years Mean age: 38 years Mean BMI: 29.91 kg/m^2^	Control Group (n = 16) 25 g/d of quinoa seed (n = 16) 50 g/d of quinoa seed (n = 18)	12 weeks	↓ TG in the 50 g/d quinoa group. No changes in BMI, TC, and LDL-C While the prevalence of metabolic syndrome in the control group increased by 6.8%, it decreased in the 25 g/d quinoa group by 41% and in the 50 g quinoa group by 70%
[59]	3542 participants in China Age > 15 years	No intervention Food intake evaluation with a food frequency questionnaire	-	↓ TC and LDL-C routinely buckwheat consuming group. ↓ Prevalences of hypertriglyceridemia and hyperlipidemia
[60]	850 participants in China Aged from 15 to 77 years	No intervention Food intake evaluation with food records	-	↓ Blood pressure 100 g/d or higher buckwheat consumption is associated with lower LDL-C, TC levels, and a higher HDL/TC ratio
[61]	Phase I: 7 healthy males aged from 18 to 22 years Phase II: 5 healthy males for OGTT	Replacement of traditional cereal with 100 g of buckwheat bread	4 weeks	↑ HDL-C and HDL/TC ratio No change in fasting glucose or OGTT in the 5 subjects in phase II
[62]	20 patients receiving statin therapy (F:13, M:7) Mean age: 59.45 years	300 g/d of buckwheat-enriched wheat bread 300 g/d of wheat bread	4 weeks	↓ TC, LDL-C, and LDL-C/HDL-C
[63]	62 healthy women Mean age: 46 years Mean BMI: 25.5 kg/m^2^	*Fagopyrum tataricum* containing 360 mg equivalent of rutin (n = 30) *Fagopyrum esculentum* containing 17 mg equivalent of rutin (n = 32)	2 weeks	↓ TC and HDL-C in all groups compared to baseline ↑ FVC of lung function ↓ Serum MPO in the *Fagopyrum tataricum* group
[64]	38 people with hypercholesterolemia (F:21, M:17) BMI between 20 and 35 kg/m^2^	80 g/d of buckwheat-enriched high-protein porridge (n = 12) 80 g/d of corn-based high-protein porridge (n = 11) 80 g/d of corn-based non-protein porridge (n = 11)	1 week	↓ TC, LDL-C, TG, and uric acid in group ↑ HDL-C, fat-free mass, and adiponectin levels
[65]	165 T2DM patients (F:98, M:67) Mean age:57 years in the buckwheat group Mean age:56.7 years in the control group	Only nutrition plan (n = 80) 150 g/day of buckwheat (n = 85)	4 weeks	↓ TC and LDL-C ↓ Insulin, insulin resistance if buckwheat intake > 110 g/d, No significant differences in blood glucose or HbA1C levels
[66]	125 people had a combined diagnosis of coronary heart disease, hypertension, and obesity (F:110, M:15) Aged from 32 to 68 years	Control: Anti-atherogenic diet (n = 40) I1: Anti-atherogenic diet + 3 g/d amaranth oil (n = 25) I2: Anti-atherogenic diet + 6 g/d amaranth oil (n = 20) I3: Anti-atherogenic diet + 12 g/d amaranth oil (n = 20) I4: Anti-atherogenic diet + 18 g/d amaranth oil (n = 20)	3 weeks	↓ Dose-dependent TG, TC, LDL, and VLDL-C ↓ Blood pressure in all groups compared to baseline 18 mL/d of amaranth oil supplementation showed the best health-promoting effect Slight improvement in MDA, GSH, GPX, SOD, and CAT
[67]	44 people (F:32, M:12) Mean age: 49 years BMI ≥ 25 kg/m^22^	20 mL/d of amaranth oil 20 mL/d of rapeseed oil	3 weeks	↑ Adiponectin levels in the amaranth oil group No significant differences in lipid and glucose markers between groups No differences in adiponectin, ox-LDL, Apo-A1, Apo-B, and Apo-E.
[68]	44 people (F:32, M:12) Mean age:48.77 years BMI ≥ 25 kg/m^2^	20 mL/d of amaranth oil 20 mL/d of rapeseed oil	3 weeks	↑ TC and LDL-C in the amaranth oil group No significant differences in hs-CRP, Selectin, VCAM-1 No significant difference in anthropometric measurements
[69]	9 people with prediabetes (F:6; M:3) Mean age:69.6 years Mean BMI: 28.4 kg/m^2^	Diet included quinoa-based carbohydrate sources such as quinoa seed, quinoa flakes, bread, cake, biscuits, crackers, and pasta Diet included standard carbohydrate sources	4 weeks	↓ Blood glucose and HbA1c ↓ Body weight, BMI, and waist circumference ↓ Carbohydrate intake, ↑ lipid, and amino acid intake No difference in TC, LDL-C, HDL-C, TG, and blood pressure
[70]	37 healthy overweight men Mean age: 51.5 years Mean BMI: 27.7 kg/m^2^	20% quinoa flour containing bread 100% refined wheat flour for bread.	4 weeks	↓ Blood glucose and AUC for glucose levels ↓ LDL- C in both groups compared to baseline No differences in anthropometric measurements, antioxidant capacity No differences in energy and nutrient intake, except for carbohydrates
[71]	14 healthy people Aged from 20 to 50 years	100 g/d of bread containing 20% quinoa flour and 3% wheat bran	3 months	↓ TC, LDL-C, VLDL-C, and TG levels
[72]	133 people with impaired glucose tolerance (F:68, M:65) Aged from 39 to 76 years Mean BMI: 24.5 kg/m^2^	Control group (n = 69) 100 g/d of quinoa consumption (n = 64)	1 year	↓ Postprandial glucose, HbA1C, HOMA-IR ↓ TC, LDL-C ↓ BMI, waist circumference, and blood pressure The progression rate to diabetes among participants in the quinoa group is significantly lower than that observed in the control group
[73]	201 people impaired glucose tolerance Mean Age: 57.16 years	Control group (n = 69) 100 g/d of multigrain (n = 68) 100 g/d of quinoa (n = 64)	1 year	Lower conversion rate from impaired glucose tolerance to T2DM ↓ Fasting insulin ↓ HOMA-IR
[74]	26 healthy people (F:22, M:4) Mean age: 50 years Mean BMI: 24 kg/m^2^	I1: Sugary water I2: Black current I3: Fermented quinoa-based blackcurrant product I4: Fermented quinoa All include equally 31 g of available carbohydrates	Acute study (4 days)	↓ Glucose response in fermented quinoa-based blackcurrant product Fermented quinoa-based blackcurrant product provided more balanced glucose levels and prevented marginal fluctuations in postprandial glycemic and insulin levels
[75]	12 healthy people (F:6 M:6) 12 diabetic people (F:7 M:5) Mean BMI: 21.6 kg/m^2^	Buckwheat bread (n = 12) Quinoa bread (n = 12) White wheat (n = 12) All include equally 50 g of available carbohydrates	1 day (Acute study)	↓ Glycemic responses of buckwheat in healthy people ↓ AUC for glucose in buckwheat- and quinoa-consuming diabetic subjects A gradual decrease in glucose levels was observed in the buckwheat and quinoa group, while a rapid decrease was observed in the white wheat bread group
[76]	Stage 1: Healthy people (F:5, M:5) Mean Age: 25 years Stage 2: People with T2DM (F:3, M:7) Mean Age: 55 years	Buckwheat bread Wheat bread Bread is made with both buckwheat and wheat flour All include equally 50 g of available carbohydrates	2 h (Acute study)	No difference in increment on glucose between buckwheat and mixture bread ↑ Blood glucose in the white wheat bread group (Stage 2)
[77]	12 healthy people (F:6 M:6) Mean age: 37.3 years, Mean BMI: 23.5 kg/m^2^ 12 people with T2DM (F:7, M:5) Mean age: 60.8 years, Mean BMI: 32.4 kg/m^2^	Acute phase: Cracker made from buckwheat flour Cracker made from rice flour All include equally 50 g of available carbohydrates Second Phase: 1 portion of a buckwheat cracker	1 week (Second phase)	Acute phase: ↓ AUC values for GLP-1 and GIP in diabetic people No significant difference in the AUC value of glucose, insulin, and C-peptide Second phase: No significant differences in glucose, lipids, and apolipoproteins between groups
[78]	10 people with type 1 diabetes and Celiac disease (F:8, M:2) Mean age: 32 years Mean BMI: 22 kg/m^2^	Stage 1: 100 g of buckwheat pasta (50 g available carbohydrates) 60 g of corn pasta (including 50 g available carbohydrates) Stage 2: Ad libitum meal	Acute study	↓ Postprandial blood glucose levels in stage 1 ↓ AUC values for glucose
[79]	13 healthy people (F:10, M:3) Aged from 18 to 60 years BMI between 18.5 and 35 kg/m2 Mean Age: 37.3 years	Wheat pasta Pasta made from rice flour Pasta made from corn and rice flour Pasta made from corn and quinoa flour	Acute study (2 h)	↑ AUC for glucose in the group consuming pasta made from corn and rice flour No significant differences between other groups
[80]	102 diabetic people (F:61, M:41) Aged from 30 to 80 years Mean BMI: 26.84 kg/m^2^	Systematic diet plans and intensive nutritional education (n = 52) Replacement of 100 g/d of wheat or rice with 100 g/d of buckwheat (n = 52)	4 weeks	↓ UACR and UN in the buckwheat group Replacement of traditional cereals with *Tartary buckwheat* alleviated renal dysfunctions in T2DM patients
[81]	38 healthy people Aged from 18 to 50 years BMI between 18 and 25 kg/m^2^	Control group (n = 8) Snack bar (90% amaranth, 5% acha, and 5% millet) (n = 10) Snack bar (amaranth 47.98%, acha 26.68%, pearl millet 25.34%) (n = 10) Snack bar (%100 oat) (n = 10)	2 h (Acute study)	↓ Glycemic index of 90% amaranth, 5% acha, and 5% millet snack bar
[82]	81 obese people (F:51, M:30) Aged from 25 to 70 years BMI > 30 kg/m^2^	Calorie-restricted diet + physical activity + 20 mL/d of amaranth oil (n = 26) Calorie-restricted diet + physical activity + 20 mL/d of rapeseed oil (n = 26) Only calorie-restricted diet + physical activity (n = 29)	3 weeks	↓ Weight, BMI, and waist and hip circumferences, and fat mass in all groups compared to baseline ↓ Insulin and HOMA-IR in both the amaranth oil and rapeseed oil groups. ↓ Glucose, TC, TG/HDL ratio, LDL-C, and TG in the amaranth oil group
[83]	38 healthy adult males Mean age: 24,06 years Mean BMI:23.13 kg/m2	White wheat and oat bread (n = 15) White wheat spaghetti + oat spaghetti + buckwheat spaghetti (n = 14) Quinoa risotto + rice risotto (n = 9)	1 day (Acute study)	↑ Satiating efficiency indices for alternative crops compared to traditional cereal foods. Similar energy intake was recorded in an ad libitum meal
[84]	38 healthy people Aged from 20 to 70 years BMI between 18–30 kg/m2	Buckwheat groats containing 50 g of available carbohydrates Pita bread containing 50 g of available carbohydrate Corn or rice-based product containing 50 g of available carbohydrate	14 days	No significant difference in VAS appetite scores or energy consumption compared with snack products made from corn or rice flour
[85]	144 people (F:103; M:41) Mean BMI: 22.18 vs. 22.25 kg/m^2^ Mean Age: 54.58 vs. 53.66 years	80 g/d of Tartary Buckwheat noodle (n = 73) 80 g/d of Wheat noodle (n = 71)	12 weeks	↓ Ox- LDL and TBARS levels No difference in HDL-C, LDL-C, TC. No difference in Urinary 8-OHdG No difference in weight and BMI between groups No significant differences in the atherosclerosis index
[86]	19 obese people (F:6, M:13) Mean BMI = 41.1 kg/m^2^ Mean age 48.3 years	Stage 1: Calorie-restricted diet for 2 weeks Stage 2: Calorie restriction diet + 20 mL/d of canola oil (n = 11) Calorie restriction diet + 20 mL/d of amaranth oil (n = 8)	3 weeks	↓ Body weight in both groups compared to baseline ↓ Fat mass, BMR, and waist/hip ratio only in the canola oil group ↑ Oxidative status in both groups in stage 2
[87]	12 people (F:6, M:6) Aged from 18 to 25 years BMI between 18–26 kg/m^2^	High-fat/high-sugar cookie (n = 12) Quinoa cookies (7.1 g *C. quinoa*) (n = 12)	12 days	↑ Diversity in microbiota Improved bacterial composition acting in carbohydrate metabolism

Note: ↓ indicates decrease; ↑ indicates increase.

## Data Availability

No new data were created or analyzed in this study. Data sharing is not applicable to this article.

## Supplementary Materials

The following supporting information can be downloaded at: https://www.mdpi.com/article/10.3390/nu18071093/s1, Supplementary Tables can be found as Table S1: Botanical Classification, Production, Processing, Preparation, and Cooking Techniques of Pseudocereals Used in Included Studies; Table S2: The Most Significant Cardiometabolic Effects of Pseudocereals Stratified by Intervention Duration; Table S3: Cardiometabolic Effects of Pseudocereals Stratified by Intervention Duration; Table S4: Dose Ranges (g/day) of Pseudocereals Reported in Included Human Studies.

## Abbreviations

The following abbreviations are used in this manuscript:

ACE	Angiotensin Converting Enzyme
AMPK	Adenosine Monophosphate-Activated Protein Kinase
Apo-A1	Apolipoprotein A1
Apo-B	Apolipoprotein B
Apo-E	Apolipoprotein E
AUC	Area Under Curve
BMI	Body Mass Index
BMR	Basal Metabolic Rate
CAT	Catalase
DPP-IV	Dipeptidyl Peptidase-IV
FAS	Fatty Acid Synthase
FVC	Forced Vital Capacity
G6PDH	Glucose-6-Phosphate Dehydrogenase
GIP	Gastric Inhibitory Polypeptide
GLP	Glucagon-Like Peptide-1
GLUT-4	Glucose Transporter Type 4
GPX	Glutathione Peroxidase
GSH	Glutathione-S-Transferase
HbA1c	Hemoglobin A1c
HDL-C	High-Density Lipoprotein Cholesterol
HMG-CoA	3-Hydroxy-3-Methyl-Glutaryl-Coenzyme A Reductase
HOMA-IR	Homeostasis Model Assessment of Insulin Resistance
hs-CRP	High-Sensitivity C-Reactive Protein
LDL-C	Low-Density Lipoprotein Cholesterol
MDA	Malondialdehyde
MPO	Serum Myeloperoxidase
NO	Nitric Oxide
OGTT	Oral Glucose Tolerance Test
ox-LDL	Oxidized Low-Density Lipoprotein
PPAR-ɣ	Peroxisome Proliferator-Activated Receptor Gamma
RAAS	Renin-Angiotensin-Aldosterone System
ROS	Reactive Oxygen Species
SCFA	Short Chain Fatty Acid
SOD	Superoxide Dismutase
T2DM	Type 2 Diabetes Mellitus
TBAR	Thiobarbituric Acid
TC	Total Cholesterol
TG	Triglycerides
UACR	Urine Albumin-to-Creatinine Ratio
UCP	Uncoupling Protein
UN	Urea Nitrogen
VAS	Visual Analog Scale
VCAM-1	Vascular Cell Adhesion Molecule
