# Supplementary material for: A Narrative Review on Pseudocereals and Cardiometabolic Health: Biological Mechanisms and Evidence from Human Studies
Source: Nutrients. 2026 Mar 29;18(7):1093. doi: 10.3390/nu18071093 (PMC13075176; doi:10.3390/nu18071093)
Supplement: Supplementary file 1 [file nutrients-18-01093-s001.zip › Supplementary Table S1.pdf]

Supplementary Table S1. Botanical Classification, Processing, Preparation, Cooking Techniques, and Cardiometabolic Effects of Pseudocereals in Included Studies

Ref	Botanical Classification, Processing & Preparation				
	Pseudocereal	Botanical Type / Species	Processing Method	Preparation / Food Form	Cooking Technique
[55]	Quinoa	*Chenopodium quinoa*	Milling into flour	Biscuit (60 g quinoa flour/100 g)	Baked
[56]	Quinoa	*Chenopodium quinoa*	Processed into bar form	Quinoa bar (9.75 g/bar × 2)	Ready-to-eat bar
[57]	Quinoa	*Chenopodium quinoa*	Flaked	Quinoa flakes (QF)	Ready-to-eat flakes
[58]	Quinoa	*Chenopodium quinoa*	Whole seed	Whole quinoa seed (25 or 50 g/d)	Cooked (boiled/steamed)
[59]	Buckwheat	*Fagopyrum esculentum* / *Fagopyrum tataricum*	Whole grain / traditional milling	Habitual food intake (FFQ)	Routine habitual cooking
[60]	Buckwheat	*Fagopyrum esculentum* / *Fagopyrum tataricum*	Whole grain / traditional milling	Habitual food intake (food records)	Routine habitual cooking (≥100 g/d)
[61]	Buckwheat	*Fagopyrum esculentum*	Milling into flour; bread making	Buckwheat bread (100 g/d replacing traditional cereal)	Baked
[62]	Buckwheat	*Fagopyrum esculentum*	Flour enrichment of wheat bread	Buckwheat-enriched wheat bread (300 g/d)	Baked
[63]	Buckwheat	*Fagopyrum tataricum* / *F. esculentum*	Rutin extraction / standardized supplement	Standardized buckwheat extract (capsule/tablet)	Oral supplementation; no cooking
[64)	Buckwheat	*Fagopyrum esculentum*	Milling; protein enrichment	Buckwheat-enriched high-protein porridge (80 g/d)	Cooked as porridge
[65]	Buckwheat	*Fagopyrum esculentum* / *Fagopyrum tataricum*	Whole grain	Whole buckwheat grain (150 g/d)	Cooked (boiled/steamed)
[66]	Amaranth	*Amaranthus spp.*	Cold-press oil extraction	Amaranth oil (3–18 g/d)	Oral oil supplementation; no cooking
[67]	Amaranth	*Amaranthus spp.*	Cold-press oil extraction	Amaranth oil (20 mL/d)	Oral oil supplementation; no cooking
[68]	Amaranth	*Amaranthus spp.*	Cold-press oil extraction	Amaranth oil (20 mL/d)	Oral oil supplementation; no cooking
[69]	Quinoa	*Chenopodium quinoa*	Multiple: whole seed, flakes, flour, pasta	Quinoa seed, flakes, bread, cake, biscuits, crackers, pasta	Baked / boiled / mixed
[70]	Quinoa	*Chenopodium quinoa*	Milling into flour (20% substitution)	Bread (20% quinoa flour + 80% wheat flour)	Baked
[71]	Quinoa	*Chenopodium quinoa*	Milling (20% flour) + wheat bran (3%)	Bread (20% quinoa flour + 3% wheat bran, 100 g/d)	Baked
[72]	Quinoa	*Chenopodium quinoa*	Whole seed	Whole quinoa seed (100 g/d)	Cooked (boiled)
[73]	Quinoa	*Chenopodium quinoa*	Whole seed	Whole quinoa (100 g/d) or multigrain (100 g/d)	Cooked (boiled)
[74]	Quinoa	*Chenopodium quinoa*	Fermentation	Fermented quinoa-based blackcurrant drink (31 g CHO)	Fermented beverage; no cooking
[75]	Buckwheat / Quinoa	*Fagopyrum esculentum* / *Chenopodium quinoa*	Milling into flour; bread making	Buckwheat bread / quinoa bread (50 g available CHO)	Baked
[76]	Buckwheat	*Fagopyrum esculentum*	Milling into flour; bread making	Buckwheat / mixed buckwheat-wheat bread (50 g available CHO)	Baked
[77]	Buckwheat	*Fagopyrum esculentum*	Milling into flour; cracker production	Buckwheat flour cracker (50 g available CHO)	Baked / oven-dried
[78]	Buckwheat	*Fagopyrum esculentum*	Extrusion / pasta manufacturing	Buckwheat pasta (100 g; 50 g available CHO)	Boiled
[79]	Quinoa	*Chenopodium quinoa*	Milling into flour; pasta manufacturing	Pasta: corn + quinoa flour (50 g available CHO)	Boiled
[80]	Buckwheat	*Fagopyrum tataricum*	Whole grain / noodle processing	Tartary buckwheat replacing wheat or rice (100 g/d)	Cooked as noodles / boiled
[81]	Amaranth	*Amaranthus spp.*	Extrusion / snack bar production	Snack bar (90% amaranth + 5% acha + 5% millet)	Extruded / baked; ready-to-eat
[82]	Amaranth	*Amaranthus spp.*	Cold-press oil extraction	Amaranth oil (20 mL/d) + calorie-restricted diet	Oral oil supplementation; no cooking
[83]	Buckwheat / Quinoa	*Fagopyrum esculentum* / *Chenopodium quinoa*	Extrusion (spaghetti); risotto preparation	Buckwheat spaghetti / quinoa risotto (ad libitum)	Boiled / cooked as risotto
[84]	Buckwheat	*Fagopyrum esculentum*	Whole grain / groat form	Buckwheat groats (50 g available CHO)	Cooked groats
[85]	Buckwheat	*Fagopyrum tataricum*	Milling into flour; noodle manufacturing	Tartary buckwheat noodle (80 g/d)	Boiled
[86]	Amaranth	*Amaranthus spp.*	Cold-press oil extraction	Amaranth oil (20 mL/d) + calorie-restricted diet	Oral oil supplementation; no cooking

**[87]**	Quinoa	*Chenopodium quinoa*	Milling into flour; cookie baking	Quinoa cookie (7.1 g quinoa/cookie)	Baked

↓ = decrease; ↑ = increase; — = not assessed or not reported; CHO = carbohydrate; FFQ = food frequency questionnaire; OGTT = oral glucose tolerance test; T1DM = type 1 diabetes mellitus; T2DM = type 2 diabetes mellitus; TC = total cholesterol; LDL-C = low-density lipoprotein cholesterol; HDL-C = high-density lipoprotein cholesterol; TG = triglycerides; VLDL-C = very low-density lipoprotein cholesterol; HbA1c = glycated hemoglobin; HOMA-IR = homeostatic model assessment of insulin resistance; BMI = body mass index; AUC = area under the curve; Ox-LDL = oxidized LDL; GLP-1 = glucagon-like peptide-1; GIP = gastric inhibitory polypeptide; UACR = urine albumin-to-creatinine ratio; BUN = blood urea nitrogen; TBARS = thiobarbituric acid reactive substances.
