# Supplementary material for: A Narrative Review on Pseudocereals and Cardiometabolic Health: Biological Mechanisms and Evidence from Human Studies
Source: Nutrients. 2026 Mar 29;18(7):1093. doi: 10.3390/nu18071093 (PMC13075176; doi:10.3390/nu18071093)
Supplement: Supplementary file 1 [file nutrients-18-01093-s001.zip › Supplementary Table S2.pdf]

Supplementary Table S2. The Most Significant Cardiometabolic Effects of Pseudocereals

Ref	Pseudocereal	Anti-Hyperlipidemic Effects	Anti-Hyperglycemic Effects	Anti-Obesity Effects	Antihypertensive Effects
[55]	Quinoa	↓ TC, LDL-C, TC:HDL ratio	---	↓ Body weight, BMI	↓ Blood pressure
[56]	Quinoa	↓ TC, LDL-C, TG	↓ Glucose levels (men only)	↓ Body weight	↓ Blood pressure
[57]	Quinoa	↓ LDL-C, TC, TG<br>↑ GSH<br>↓ TBARS and Vitamin E	---	---	---
[58]	Quinoa	↓ TG (50 g/d group only)<br>No changes in TC and LDL-C	↓ Metabolic syndrome prevalence<br>(−41% at 25 g/d; −70% at 50 g/d)	No change in BMI	---
[59]	Buckwheat	↓ TC, LDL-C<br>↓ Prevalences of hypertriglyceridemia and hyperlipidaemia	---	---	---
[60]	Buckwheat	↓ LDL-C, TC<br>↑ HDL/TC ratio (≥100 g/d)	---	---	↓ Blood pressure
[61]	Buckwheat	↑ HDL-C, ↑ HDL/TC ratio	No change in fasting glucose or OGTT	---	---
[62]	Buckwheat	↓ TC, LDL-C, LDL-C/HDL-C ratio	---	---	---
[63]	Buckwheat	↓ TC, HDL-C (both groups vs. baseline)	---	---	---
[64]	Buckwheat	↓ TC, LDL-C, TG<br>↑ HDL-C ↓ Uric acid	---	↑ Fat-free mass<br>↑ Adiponectin	---
[65]	Buckwheat	↓ TC, LDL-C	↓ Insulin resistance (>110 g/d)<br>No significant differences in blood glucose or HbA1c	---	---
[66]	Amaranth	↓ TC, TG, LDL-C, VLDL-C (dose-dependent)	---	---	↓ Blood pressure (all groups)
[67]	Amaranth	No significant differences in lipid markers	No significant differences in glucose markers	↑ Adiponectin	---
[68]	Amaranth	↑ TC and LDL-C in amaranth oil group	---	No significant difference in anthropometric measurements	---
[69]	Quinoa	No difference in TC, LDL-C, HDL-C, TG	↓ Blood glucose, HbA1c	↓ Body weight, BMI, waist circumference	No difference in blood pressure
[70]	Quinoa	↓ LDL-C (both groups vs. baseline)	↓ Blood glucose<br>↓ AUC for glucose	No change in anthropometrics	---
[71]	Quinoa	↓ TC, LDL-C, VLDL-C, TG	---	---	---
[72]	Quinoa	↓ TC, LDL-C	↓ Postprandial glucose, HbA1c, HOMA-IR<br>↓ Progression to T2DM	↓ BMI, waist circumference	↓ Blood pressure
[73]	Quinoa	---	↓ Fasting insulin, HOMA-IR<br>Lower conversion rate to T2DM	---	---
[74]	Quinoa	---	↓ Glucose response<br>Balanced postprandial glucose and insulin levels	---	---
[75]	Buckwheat / Quinoa	---	↓ Glycemic response (buckwheat, healthy)<br>↓ AUC glucose (both, diabetic)	---	---
[76]	Buckwheat	---	↑ Blood glucose in white wheat group<br>Buckwheat attenuated glucose rise (T2DM)	---	---
[77]	Buckwheat	No difference in lipids (second phase)	↓ AUC values for GLP-1 and GIP (acute, diabetic)<br>No difference in glucose AUC	---	---
[78]	Buckwheat	---	↓ Postprandial blood glucose<br>↓ AUC for glucose (T1DM + coeliac)	---	---
[79]	Quinoa	---	↑ AUC glucose in corn+rice pasta group<br>No significant difference in quinoa pasta group	---	---
[80]	Buckwheat	---	↓ UACR and blood urea nitrogen<br>Alleviated renal dysfunction in T2DM	---	---
[81]	Amaranth	---	↓ glycemic index of 90% amaranth snack bar	---	---
[82]	Amaranth	↓ TC, TG, LDL-C<br>↓ TG/HDL ratio	↓ Glucose, insulin, HOMA-IR	↓ Weight, BMI, waist/hip circumference, fat mass	---
[83]	Buckwheat / Quinoa	---	↑ Satiety indices for pseudocereal foods<br>(vs. wheat)	---	---
[84]	Buckwheat	---	No significant difference in appetite or energy intake	---	---
[85]	Buckwheat	No difference in HDL-C, LDL-C, TC<br>↓ Ox-LDL and TBARS	---	No difference in weight or BMI	---
[86]	Amaranth	---	---	↓ Body weight<br>↓ Fat mass	---
[87]	Quinoa	---	Improved microbiota in carbohydrate metabolism	---	---

↓ = decrease; ↑ = increase; --- = not assessed or not reported; T2DM = type 2 diabetes mellitus; T1DM = type 1 diabetes mellitus; TC = total cholesterol; LDL-C = low-density lipoprotein cholesterol; HDL-C = high-density lipoprotein cholesterol; TG = triglycerides; VLDL-C = very low-density lipoprotein cholesterol; HbA1c = glycated hemoglobin; HOMA-IR = homeostatic model assessment of insulin resistance; BMI = body mass index; AUC = area under the curve; Ox-LDL = oxidized LDL; GLP-1 = glucagon-like peptide-1; GIP = gastric inhibitory polypeptide; UACR = urine albumin-to-creatinine ratio; OGTT = oral glucose tolerance test; TBARS = thiobarbituric acid reactive substances; GSH = glutathione; MPO = myeloperoxidase; BMR = basal metabolic rate; UN = urea nitrogen.
