# Supplementary material for: A Narrative Review on Pseudocereals and Cardiometabolic Health: Biological Mechanisms and Evidence from Human Studies
Source: Nutrients. 2026 Mar 29;18(7):1093. doi: 10.3390/nu18071093 (PMC13075176; doi:10.3390/nu18071093)
Supplement: Supplementary file 1 [file nutrients-18-01093-s001.zip › Supplementary Table S3.pdf]

Supplementary Table S3. Cardiometabolic Effects of Pseudocereals Stratified by Intervention Duration

Duration	Ref	Pseudocereal	Dose	Key Cardiometabolic Findings
**Acute / Short-Term Studies (< 4 Weeks)**				
	[64]	Buckwheat	80 g/d porridge (1 week)	↓ TC, LDL-C, TG; ↑ HDL-C; ↑ adiponectin; ↓ uric acid
—	[66]	Amaranth	3–18 g/d oil (3 weeks)	↓ TC, LDL-C, TG, VLDL-C (dose-dependent); ↓ blood pressure
—	[67]	Amaranth	20 mL/d oil (3 weeks)	↑ Adiponectin; no significant lipid differences
—	[68]	Amaranth	20 mL/d oil (3 weeks)	↑ TC and LDL-C; no change in inflammatory or anthropometric markers
—	[74]	Quinoa	Fermented drink, 31 g CHO (4 days)	↓ Glucose response; more balanced postprandial glucose and insulin levels
—	[75]	Buckwheat / Quinoa	50 g available CHO (1 day)	↓ glycemic response; ↓ AUC glucose in diabetic subjects
—	[76]	Buckwheat	50 g available CHO (2 hours)	Attenuated postprandial glucose rise vs. white wheat (T2DM)
—	[77]	Buckwheat	50 g available CHO (acute phase)	↓ AUC for GLP-1 and GIP in diabetic subjects
—	[78]	Buckwheat	100 g pasta (50 g available CHO, acute)	↓ Postprandial blood glucose; ↓ AUC for glucose (T1DM + coeliac)
—	[79]	Quinoa	50 g available CHO (2 hours)	No significant glucose difference for quinoa pasta vs. wheat
—	[81]	Amaranth	~30–40 g snack bar (2 hours)	↓ glycemic index of 90% amaranth snack bar
—	[82]	Amaranth	20 mL/d oil (3 weeks)	↓ TC, TG, LDL-C, glucose, insulin, HOMA-IR; ↓ weight, BMI
—	[83]	Buckwheat / Quinoa	Ad libitum (1 day)	↑ Satiating efficiency indices for pseudocereal foods
—	[84]	Buckwheat	50 g available CHO (14 days)	No significant difference in appetite or energy intake
—	[86]	Amaranth	20 mL/d oil (3 weeks)	↓ Body weight; ↓ fat mass only in canola oil group
—	[87]	Quinoa	7.1 g quinoa/cookie (12 days)	↑ Microbiota diversity; improved carbohydrate-metabolizing bacteria
**Medium-Term Studies (4–12 Weeks)**				
**4–12 Weeks**	[55]	Quinoa	15 g/d biscuit (28 days)	↓ TC, LDL-C, TC: HDL; ↓ body weight, BMI; ↓ blood pressure
—	[56]	Quinoa	19.5 g/d bar (30 days)	↓ TC, LDL-C, TG; ↓ glucose (men); ↓ body weight; ↓ blood pressure
—	[57]	Quinoa	25 g/d flakes (4 weeks)	↓ LDL-C, TC, TG; ↑ GSH; ↑ urinary enterolignans
—	[58]	Quinoa	25 or 50 g/d seed (12 weeks)	↓ TG (50 g/d); ↓ metabolic syndrome prevalence
—	[61]	Buckwheat	100 g/d bread (4 weeks)	↑ HDL-C; ↑ HDL/TC ratio; no change in glucose or OGTT
—	[62]	Buckwheat	300 g/d of enriched bread (4 weeks)	↓ TC, LDL-C, LDL-C/HDL-C ratio
—	[63]	Buckwheat	Rutin extract (2 weeks)	↓ TC; ↓ serum MPO (Tartary type); ↑ lung FVC
—	[65]	Buckwheat	150 g/d whole grain (4 weeks)	↓ TC, LDL-C; ↓ insulin resistance (>110 g/d)
—	[69]	Quinoa	~100 g/d mixed quinoa forms (4 weeks)	↓ Blood glucose, HbA1c; ↓ weight, BMI, waist circumference
—	[70]	Quinoa	Bread with 20% quinoa flour (4 weeks)	↓ Blood glucose AUC; ↓ LDL-C
—	[77]	Buckwheat	One portion cracker/d (One week)	No significant differences in glucose or lipid markers
—	[80]	Buckwheat	100 g/d Tartary buckwheat (4 weeks)	↓ UACR, BUN; alleviated renal dysfunction in T2DM
—	[85]	Buckwheat	80 g/d Tartary buckwheat noodle (12 weeks)	↓ Ox-LDL, TBARS; no significant change in standard lipids or BMI
**Long-Term Studies (> 12 Weeks)**				
**> 12 Weeks**	[71]	Quinoa	100 g/d bread (3 months)	↓ TC, LDL-C, VLDL-C, TG
—	[72]	Quinoa	100 g/d whole seed (1 year)	↓ Postprandial glucose, HbA1c, HOMA-IR; ↓ TC, LDL-C; ↓ BMI; ↓ blood pressure; ↓ T2DM progression
—	[73]	Quinoa	100 g/d whole seed (1 year)	↓ Fasting insulin, HOMA-IR; lower T2DM conversion rate

Studies stratified into three duration categories: acute/short-term (< 4 weeks), medium-term (4–12 weeks), and long-term (> 12 weeks). ↓ = decrease; ↑ = increase; T1DM = type 1 diabetes mellitus; T2DM = type 2 diabetes mellitus; OGTT = oral glucose tolerance test; AUC = area under the curve; GLP-1 = glucagon-like peptide-1; GIP = gastric inhibitory polypeptide; BMI = body mass index; HOMA-IR = homeostatic model assessment of insulin resistance; UACR = urine albumin-to-creatinine ratio; BUN = blood urea nitrogen; Ox-LDL = oxidized LDL; TBARS = thiobarbituric acid reactive substances; FVC = forced vital capacity; GSH = glutathione; MPO = myeloperoxidase.
