# Supplementary material for: A Narrative Review on Pseudocereals and Cardiometabolic Health: Biological Mechanisms and Evidence from Human Studies
Source: Nutrients. 2026 Mar 29;18(7):1093. doi: 10.3390/nu18071093 (PMC13075176; doi:10.3390/nu18071093)
Supplement: Supplementary file 1 [file nutrients-18-01093-s001.zip › Supplementary Table S4.pdf]

Supplementary Table S4. Dose Ranges of Pseudocereals Reported in Included Human Studies

Ref	Pseudocereal	Food / Supplement Form	Dose Range (g/day)	Key Outcome at Reported Dose
**[55]**	Quinoa	Biscuit (60% quinoa flour)	15	↓ TC, LDL-C, body weight, blood pressure
**[56]**	Quinoa	Bar (9.75 g quinoa/bar × 2 bars)	19.5	↓ TC, LDL-C, TG, body weight, blood pressure
**[57]**	Quinoa	Flakes	25	↓ LDL-C, TC, TG
**[58]**	Quinoa	Whole seed (low dose)	25	↓ Metabolic syndrome prevalence by 41%
**[58]**	Quinoa	Whole seed (high dose)	50	↓ TG; ↓ metabolic syndrome prevalence by 70%
**[69]**	Quinoa	Mixed quinoa-based carbohydrate diet	~100 (total CHO sources)	↓ Blood glucose, HbA1c, body weight, BMI
**[70]**	Quinoa	Bread (20% quinoa flour)	~30–40 (flour equivalent)	↓ Blood glucose AUC, ↓ LDL-C
**[71]**	Quinoa	Bread (20% flour + 3% wheat bran)	100 (bread weight)	↓ TC, LDL-C, VLDL-C, TG
**[72]**	Quinoa	Whole seed	100	↓ Postprandial glucose, HbA1c, HOMA-IR, TC, LDL-C, BMI, blood pressure
**[73]**	Quinoa	Whole seed	100	↓ Fasting insulin, HOMA-IR; lower T2DM conversion rate
**[61]**	Buckwheat	Bread (100% buckwheat flour)	100 (bread weight)	↑ HDL-C and HDL/TC ratio
**[62]**	Buckwheat	Wheat bread enriched with buckwheat flour	300 (bread weight)	↓ TC, LDL-C, LDL-C/HDL-C
**[63]**	Buckwheat	Standardized extract (rutin equiv. 360 mg)	~1–2 (extract)	↓ TC, serum MPO (Tartary type)
**[64]**	Buckwheat	High-protein porridge	80	↓ TC, LDL-C, TG; ↑ HDL-C
**[65]**	Buckwheat	Whole grain	150	↓ TC, LDL-C; ↓ insulin resistance (>110 g/d)
**[80]**	Buckwheat	Tartary buckwheat replacing wheat/rice	100	↓ UACR and BUN; alleviated renal dysfunction (T2DM)
**[85]**	Buckwheat	Tartary buckwheat noodle	80	↓ Ox-LDL, TBARS; no change in standard lipids
**[66]**	Amaranth	Oil supplement (low dose)	3	↓ TC, LDL-C, TG, VLDL-C (dose-dependent)
**[66]**	Amaranth	Oil supplement (medium dose)	6	↓ TC, LDL-C, TG, VLDL-C
**[66]**	Amaranth	Oil supplement (medium-high dose)	12	↓ TC, LDL-C, TG, VLDL-C
**[66]**	Amaranth	Oil supplement (high dose)	18	Best cardiometabolic effect; ↓ blood pressure
**[67]**	Amaranth	Oil supplement	20 mL (~18 g)	↑ Adiponectin; no significant lipid change
**[68]**	Amaranth	Oil supplement	20 mL (~18 g)	↑ TC and LDL-C in amaranth oil group
**[82]**	Amaranth	Oil + calorie-restricted diet + exercise	20 mL (~18 g)	↓ TC, TG, LDL-C, glucose, insulin, HOMA-IR; ↓ weight, BMI
**[86]**	Amaranth	Oil + calorie-restricted diet	20 mL (~18 g)	↓ Body weight; ↓ fat mass (canola group only)
**[81]**	Amaranth	Snack bar (90% amaranth)	~30–40 (bar weight)	↓ Glycemic index

Doses represent the pseudocereal ingredient or supplement amount per day as reported by the study authors. Amaranth oil doses are reported as mL/day; approximate gram equivalents are provided in parentheses (density ≈ 0.9 g/mL).

↓ = decrease; ↑ = increase; — = not assessed or not reported; CHO = carbohydrate; FFQ = food frequency questionnaire; OGTT = oral glucose tolerance test; T1DM = type 1 diabetes mellitus; T2DM = type 2 diabetes mellitus; TC = total cholesterol; LDL-C = low-density lipoprotein cholesterol; HDL-C = high-density lipoprotein cholesterol; TG = triglycerides; VLDL-C = very low-density lipoprotein cholesterol; HbA1c = glycated hemoglobin; HOMA-IR = homeostatic model assessment of insulin resistance; BMI = body mass index; AUC = area under the curve; Ox-LDL = oxidized LDL; GLP-1 = glucagon-like peptide-1; GIP = gastric inhibitory polypeptide;; BUN = blood urea nitrogen; TBARS = thiobarbituric acid reactive substances.
